# The informal way to success or failure? Findings from a comparative case study on video consultation training and implementation in two Danish hospitals

**DOI:** 10.1186/s12913-023-10163-w

**Published:** 2023-10-21

**Authors:** Susanne Eriksen, Anne Marie Dahler, Christine Øye

**Affiliations:** 1 Faculty of Health and Social Science, Western Norway University of Applied Sciences, Bjørnsonsgate 45, 5528 Haugesund, Norway; 2 Centre for Applied Welfare Research, UCL University College, Niels Bohrs Allé 1, 5230 Odense M, Denmark; 3 Faculty of Health and Social Science, Western Norway University of Applied Sciences, Klingenbergvegen 4, 5414 Stord, Norway

**Keywords:** Workplace learning, Training, Implementation, Telehealth, Video consultations

## Abstract

**Background:**

This study evaluated an attempt to implement video consultations through a novel education intervention in telehealth training and implementation in two middle-sized hospitals in Denmark. Three units tested the education intervention along with a regional decision to strengthen multidisciplinary and cross-sectoral collaboration through technology to improve service delivery by making the process more coherent and saving time and resources. This study aims to identify what contextual factors enable workplace learning, skills acquisition, and utilization of new digital skills to use and routinize video consultations in workplace practice.

**Methods:**

This qualitative case study draws on the principles of the realist evaluation framework using cross-case comparisons to test and refine program theories by exploring the complex and dynamic interaction among context, mechanism, and outcome. The methods in this study include participant observations, document analysis, semi-structured individual interviews, and focus groups. We performed an interpretive cross-case analysis, which explored the context-mechanism-outcome relationship using the guiding question, “What works, for whom, under what circumstances, and why?”.

**Results:**

Two broad mechanisms appeared to enable skills acquisition and routinization of video consultations: informal workplace learning and adjusting video consultations to professional judgment. The three units had different approaches to the implementation and training and, as such, had different outcomes. First, the skills acquired in the units differed; therefore, how and with whom they used video consultations varied. Second, video consultation use was more likely to be adjusted to workflows if unit managers were responsive to staff’s professional judgments regarding patients, as was evident in all three units.

**Conclusion:**

Our study shows that a formal training course alone is insufficient to provide healthcare professionals with the skills needed to use video consultations in workplace practice. Informal workplace learning with support on the spot and continuous follow-up seems to equip healthcare professionals with the skills to use video consultations. Video consultations are more likely to be used confidently if novel workflows are adjusted to health care professionals' knowledge, skills, and judgment and their concerns regarding patient soundness.

**Supplementary Information:**

The online version contains supplementary material available at 10.1186/s12913-023-10163-w.

## Background

Video consultations are one of many telehealth solutions used to exchange medical information through electronic communication between healthcare professionals, patients, and next of kin to improve patient outcomes, reduce travel and costs, improve communication, decrease waiting time and increase accessibility [1, 2]. Due to social distancing measures, video consultations between healthcare professionals and patients have increased during the last couple of years, especially during the COVID-19 pandemic [3, 4]. The pandemic was seen as “an opportunity in crisis” to achieve a more scaled-up and widespread implementation of video consultations in health care to meet the projected shortages in the health workforce and the changing needs of ageing populations more cost-effectively and sustainably [4].

Despite a strong policy push to use video consultations [5] and evidence suggesting that video consultations are acceptable, safe, and effective in selected conditions and settings [6–9], those implementing video consultations have faced significant organizational and infrastructural challenges [4, 10]. The most commonly reported challenges are the establishment of new workflows and organizational routines, lack of technical support, lack of resources, and lack of training [11, 12]. According to previous research, top barriers are technology-specific and could be overcome by training, change-management techniques, and alternating care delivery [12].

However, in-depth investigations of training measures in the video consultation domain are sparse. Implementing electronic health records can teach important lessons for integrating telehealth, particularly the importance of training to enhance productivity, quality, and safety [1]. Effective training measures for electronic health records recommend a combination of training methods. However, studies including detailed information on training content are lacking [13]. We also identified a report on the effectiveness of training programs in various telehealth solutions in hospitals and home healthcare services. The report identified significant gaps in the research, as only ten studies describing the design and conduct of telehealth training programs were identified. Nevertheless, the report recommends that training programs work best if the training is ongoing, contextualized and tightly coupled with practice [14]. These recommendations align with a study on virtual visits in home healthcare services. The study reports that training from healthcare professionals’ perspectives should be structured, needs-based, practical, and hands-on to enable the knowledge, skills, and attitudes required for new ways of working [15].

Working in new ways with telehealth is not a simple transition [16]. A study on cross-sectoral video consultations showed how new roles, tasks, responsibilities, and professional knowledge were transformed through a complex negotiation process between municipal—and specialized hospital nurses [17]. Moreover, the same study found that to replace the sensuous and bodily impressions available in physical consultations, the nurses performed ‘detective work’ to synthesize and make sense of decontextualized fragments of information on patients and their medical condition provided through telehealth [17]. Further, changing existing workflows requires time to train new workflows and techniques, often affecting both the efficiency and effectiveness of care and leading to resistance [12].

In summary, previous studies report that training is necessary to ensure effective and valuable use of telehealth and that telehealth-related training is essentially about learning a workplace practice skill and, as such, learning how to use telehealth concerns technical skills and adjusting to novel tasks, workflows, and roles. Therefore, it is helpful to investigate training in video consultations through the lens of workplace learning (WPL).

Policies on the digital transformation of health systems support the promotion of adequately designed training programs, including greater recognition of on-the-job-practical training measures as an efficient approach to providing healthcare professionals with new skills [18, 19]. Previous research on WPL outside health care report that 60–90% of work-related learning occurs informally [20, 21] and that the workplace offers learning outcomes that cannot be obtained in formal courses [22–24]. Moreover, research shows that people learn from each other by finding solutions for their day-to-day problems at the workplace [25–27]. Studies on WPL in health care have found that large clinical and administrative workloads are the primary barrier to WPL [28]. The main enabling factors, however, are the feeling of confidence among healthcare professionals [29], the presence of work challenges [29], management- and organizational support [28, 29], and having access to peers, expertise and learning networks [28].

The literature on video consultations is growing rapidly across several clinical areas [11]. However, we still have a limited understanding of what kind of training and skills healthcare professionals need to adopt video consultations and how to integrate new skills with existing workflows and organizational routines. As such, WPL in the video consultation domain is yet to be highlighted in more detail and considered relevant to provide healthcare professionals with the skills they need to use and routinize video consultations in workplace practice. However, we acknowledge that the implementation of video consultations is a complex social intervention [30, 31], and that interventions, such as an education intervention, may work differently in different situations and settings [32].

To investigate this, we will employ a realist evaluation (RE) [32] on an attempt to implement video consultations in three different units in two middle-sized Danish hospitals, to delve into the relationship over time between “context”, “mechanisms”, and “outcomes”. Within a realist analysis, this relationship is not seen as fixed. Rather, specific preconditions are viewed as giving rise to what realists refer to as ‘conditional causality’, implying that interventions are only effective under specific circumstances. Hence, this study aims to identify the conditions to answer the following research question: Which mechanisms work in which settings, and what contextual factors constrain and enable workplace learning, skills acquisition and the utilization of new digital skills to use and routinize video consultations?

## Methods

This study builds on a qualitative case study drawing on the principles of the RE framework [32] using cross-case comparisons. We found cross-case comparisons helpful in illuminating diversity in the implementation process [33]. RE uses program theories to explain how a program or intervention is expected to work. The program theories guide the evaluator’s search for evidence that enables the program theories to be refined and tested. The terms context (C), mechanism (M), and outcome (O) are used during the process of refining and testing the program theory. RE are beneficial when evaluating complex social interventions within complex social systems [34]. Therefore, we chose to use RE as a methodology since learning how to use technology results from complex interactions during practice, where contextual factors trigger mechanisms to generate different outcomes, such as healthcare professionals' behaviour [34]. To understand the process of implementing video consultations through an education intervention in healthcare, we needed to explore the links between C, M, and O, so-called CMO-configurations. Being underpinned by a realist philosophy of science, we assume that social systems and structures are real and that human actors respond differently to interventions in different circumstances [32]. We have, therefore, drawn on several data sources and methods to capture the complex and dynamic nature of the implementation effort unfolding over time. These methods include semi-structured individual interviews, focus groups, participatory observations, and scrutiny of documents.

### Setting

The study was conducted in two middle-sized hospitals in Denmark alongside an EU innovation project called ‘Digital and Skills Helix in Health’ (DISH). The hospitals were some of several healthcare facilities testing the novel education intervention developed in the DISH project, as there was a regional decision to strengthen the multidisciplinary and cross-sectoral collaboration with technology. The intention was that technology should be used when discharging vulnerable patients with complex diseases from hospital to municipal care, and the technology should make the process more efficient. The aim was that 30% of consultations and meetings should be virtual, and the objective of using the education intervention at the hospitals was to train staff in using video consultations for discharge planning conferences. We defined a case as a unit involved in the training and implementation of video consultations through a novel education intervention. The three cases were a neonatal unit, a cardiology unit, and a neurology unit.

### The education intervention and the concepts

The concepts in the education intervention were developed as a part of the DISH project, an EU innovation project comprising seven work packages through which the concepts were developed, tested and evaluated. The first phase of developing the concepts was a needs assessment based on a scoping review that assessed existing digital skills education programs for healthcare staff. One of the triple helix working groups in the DISH project performed this scoping review. The scoping review found that acquiring digital skills is complex and context-dependent and should not be considered in a general and singular way. Therefore, the development of the concepts considered that the concepts should not be standardized and allow openness and flexibility to adapt to the respective contexts.

The next phase of the concept development was several workshops where the triple helix partners (consisting of education institutions, telehealth clusters and healthcare organisations) in the DISH project collaborated on designing the concepts based on the needs assessment, own experiences, and also influenced by Kotter’s eight steps to change model [35]. The process eventually resulted in three concepts: preparation, on-the-job training, and assessment. The focus of this paper revolves around the preparation and on-the-job training concepts.

First, the preparation concept consisted of a framework with organizational tasks and tasks related to health care professionals. The tasks were organized into the following eight domains: Objectives, actions, stakeholder involvement, resources, technology adoption, collaboration, costs, and benefits. The preparation concept should help the facilitators to prepare the training and implementation in close collaboration with several levels of management, coordinators and IT staff, and system experts across the hospitals and the municipality. The intention with the preparation concept was to prepare the unit management for the training and implementation, to clarify needs, and to create a mutual understanding through a process characterized by shared decision-making.

Second, the on-the-job training concept guided the translation of training needs into concrete training objectives and outcomes through various questions, e.g., value, advantages, barriers, support, involvement, follow-up, and superusers. On-the-job training often used interchangeably with WPL, is characterized as acquiring knowledge or skills in the workplace by formal and informal means [36]. As such, the training could take place in units or simulation facilities. Attendees could be a mix of staff from both sectors or one-on-one training, and the content could be a mix of presentations and hands-on training. An online platform was used during the training for preparation, reflection, assignments, tasks, and evaluation. Tasks could be making a video to demonstrate the use of the technology, uploading pictures to show the strengths of the technology or group discussions on challenges regarding the implementation. The attendees received a formal course diploma after finishing the training course.

After the concepts were developed, several healthcare organizations implemented and tested them within the triple helix partnerships. The DISH project also evaluated the testing and implementation of the concepts; however, this evaluation was not research-based. Our study and the RE were initiated when the testing and implementation phase began. As the concepts were not validated or standardized, and since the concepts were flexible and about adjustments to the respective context, we wanted to find out how the ‘outcomes’ were shaped, enabled, and constrained by the interaction between the context of the education intervention and the change mechanisms. To validate the identified outcomes, we engaged with stakeholders, including DISH project participants, ‘education intervention’ developers, implementers, trainers, project leaders and assistants. Their perspectives have provided valuable insights into the relevance and accuracy of the outcomes.

### Data collection

Data were collected from August 2020 to February 2022 (see Table 1), applying a range of data sources: 1) semi-structured interviews with leaders, managers, and staff, 2) focus groups with policymakers, the director of the hospitals, managers, and staff 3) field notes from participant observations during training sessions, and 4) document analyses. The study included participant observations over a total of 5 days. The observations were conducted during five training sessions lasting 4 to 7 h. During the testing phase of the education intervention, we conducted semi-structured individual interviews, focus groups, and participant observations. The observations occurred during training, where the observer participated and observed questions, discussions, and task-solving. After the testing phase, we conducted semi-structured individual interviews and focus groups. Interviews and participant observations during the testing phase were conducted in person by AMD. After the testing phase, the interviews were conducted in person or online (using Zoom or Cisco Webex Meetings) by SE and either CØ or AMD. The same interview guide was used during all interviews. Interviews were audio-recorded and transcribed verbatim. The data collection ended when data saturation was reached. The document analyses of DISH project reports were primarily used for background information and case building.

**Table 1:** Overview of data collection

Data collection	*N* =	Role of respondents	Type of method	Duration	Researcher	Conducted
Observation 1	15	Municipal nurses (6) Hospital nurses (6) Trainers (2) Hospital IT specialist (1)	Participant observation	7 h	AMD	August 2020
Observation 2	11	Hospital nurses (8) Trainers (2) Hospital IT specialist (1)	Participant observation	7 h	AMD	November 2020
Observation 3	15	Municipal nurses (6) Hospital nurses (6) Trainers (2) Hospital IT specialist (1)	Participant observation	7 h	AMD	November 2020
Observation 4	11	Hospital nurses (8) Trainers (2) Hospital IT specialist (1)	Participant observation	7 h	AMD	March 2021
Observation 5	6	Hospital nurses (3) Trainers (2) Hospital IT specialist (1)	Participant observation	4 h	AMD	May 2021
Interview 1	3	Concept developers	Focus group	1 h 34 min	SE, CØ	September 2021
Interview 2	1	Municipal nurse	Individual interview	30 min	AMD	October 2020
Interview 3	1	Nurse	Individual interview	30 min	AMD	October 2020
Interview 4	1	Nurse	Individual interview	30 min	AMD	December 2021
Interview 5	3	Trainers	Focus group	53 min	SE, AMD	November 2021
Interview 6	3	Unit manager Nurses (2)	Focus group	1 h 21 min	SE, AMD	November 2021
Interview 7	1	Nurse	Individual interview	47 min	SE, CØ	November 2021
Interview 8	1	Unit manager	Individual interview	40 min	SE, CØ	November 2021
Interview 9	4	Municipal leaders (3) Municipal IT specialist	Focus group	57 min	SE, CØ	November 2021
Interview 10	4	Municipal middle managers (2) Municipal case worker Telehealth consultant	Focus group	41 min	SE, CØ	November 2021
Interview 11	3	Hospital director Policymakers (2)	Focus group	53 min	SE, AMD	November 2021
Interview 12	2	Unit manager Nurse	Focus group	46 min	SE, AMD	November 2021
Interview 13	2	Nurses (2)	Focus group	55 min	SE, AMD	November 2021
Interview 14	1	Concept developer	Individual interview	51 min	SE	February 2022

### Participants

We conducted 14 interviews with 30 purposively sampled respondents (see Table 1) who either contributed to the development of the concepts in the education intervention or participated in the planning of the training sessions, attended the training sessions, or provided follow-up and support after the training. Respondents from the hospitals included nurses (8), unit managers (3), trainers (3), and the director of the hospitals (1). Respondents from the municipality included middle managers (2), executive managers (3), a case worker (1), a nurse (1), an IT consultant (1), and a telehealth consultant (1). The respondents also included regional policymakers (2). An essential tenet of realist evaluation is explicitly making assumptions about the ‘program developers’ [37]. As such, four of the concept developers were also interviewed. 27 respondents were women, and 3 were men. To recruit the respondents, we contacted those responsible for the hospital training. One of the trainers became our main point of contact, and with help from the trainer, training ‘planners’ and attendees were invited to participate. The respondents received informed consents, including an overview of the study project.

### Analyses

In line with a realist analysis of data [32], we attempted to identify prominent recurrent patterns of contexts and outcomes (demi-regularities) in the data. Then, we sought to explain these through the occurring mechanisms. A cross-case analysis was used to determine how the same mechanism or sub-mechanism played out in different contexts and produced different outcomes, thereby allowing inferences about the generative causality of different contexts [33].

We understand mechanisms by the following three features: 1) mechanisms are underlying and hence often unobservable or hidden [38], 2) mechanisms are sensitive to variations in context, as well as to the operation of other mechanisms in a particular context [38], and 3) mechanisms generate outcomes, and as the mechanisms are unobservable, the causation of the outcome is inferred by examining patterns of regular contingent relations between events [39]. During data categorization, C, M, and O coding for each case were done in a coding process using the NVivo 12 software.

As deriving realist conclusions about the underlying causality of specific CMO configurations is not necessarily a straightforward exercise [40], we have strived for inter-rater reliability in the coding process through independent reading of transcripts and in the forming of initial themes, followed by discussion meetings and consensus meetings among the coders. Researcher reflexivity has been pursued through various means, aligning with qualitative research standards [41]. First, by supplementing and contesting each other’s viewpoints. Second, by acknowledging our initial assumptions through discussions about personal and professional backgrounds in the early research process to prevent conflating preconceived notions with newly acquired data insights.

## Results

The education intervention resulted from an EU innovation project involving triple helix partners from six different European countries lasting for four years (including 26 months of developing the concepts and 18 months of testing them). The three hospital units drew on several approaches to learning, including formal courses in simulation facilities, formal courses in the units, and informal learning. New approaches emerged as the implementation effort unfolded, and others fizzled out. For example, support and repetitive follow-up meetings and training were added along the way, as the trainers were unsure whether the healthcare professionals used video consultations.

We found that two broad mechanisms, each comprising several sub-mechanisms, were evident in the implementation effort. The education intervention unfolded slightly differently across the three units due to organizational structures, routines, and existing workflows across units and sectors, the characteristics and the circumstances of the patient groups and next of kin involved, and the experienced level of responsibility among health care professionals. Taking these and other contextual factors into account, we next present the three cases (including the most prominent quotes), followed by a presentation of the key enabling and constraining factors using the two mechanisms as illustrative examples to demonstrate what appeared to make each mechanism more or less likely to produce the outcome.

### Neonatal unit

A neonatal unit is for premature infants and sick or vulnerable newborns with special needs. The staff is trained in caring for and treating premature infants and sick newborns, e.g., helping babies breathe and eat. At the end of the hospitalization period, the unit offers ‘early homestay’ to babies who still need help eating but are otherwise healthy. The parents receive the needed counselling before being discharged to early homestay, and they have consultations with ‘early homestay nurses’ from the unit two times a week. Until COVID-19, these consultations were physical home visits. Due to the COVID-19 pandemic social distancing measures, home visits were no longer possible. Video consultations were therefore proposed to replace these visits. An added value of replacing physical visits with video consultations was the opportunity to save time and resources that could be used in the unit instead. In line with the regional decision to increase the number of video consultations, it was decided that video consultation use should continue even though the social distancing measures were removed. Additionally, the two weekly consultations were reduced to one consultation per week.

Before implementing the new workflow, a one-hour preparation meeting was held between the trainers, the unit management, and an IT specialist to plan the training and solve tasks concerning management, workflows, training attendees’ needs, and the value of the training. The training participants were three nurses and one IT specialist from the unit who received two half-day training sessions in the hospitals’ simulation facilities. Additionally, one nurse received informal follow-up training and support the first couple of times she used the video consultations.

Through the participant observations of the neonatal unit training session, we got an insight into the training content, which consisted of practical information, hands-on training, troubleshooting, and questions. The hands-on training was based on a case where the trainer took on the role of a parent. The trainer, located in another room, was invited to a video consultation by the nurses. The trainer’s arm was playing the role of the baby, and the nurses asked her to move the camera around so they could detect skin details. During lunch, the trainer sneaked into the training facility and turned off sound and camera or placed black tape on the lens of devices used by the nurses. After lunch, the participants had to ‘trouble-shoot’ and make their devices work again.

The participants expressed that they achieved the digital skills they needed for video consultations and appreciated the possibility of support and follow-up training. The trainers added follow-up training 30–60-90 and 180 days after the initial training course as they were unsure whether the video consultations were used. According to one of the trainers, *“That’s where the missing link is. It’s the transition from the training course to the integration in the unit. I can see that we can do something more there. Develop or adjust what we already do.* […] *We have good experience, and it works really well if they first attended our training course, and then we come out there* [to the unit]*. They* [the staff] *appreciate that we come to them and help them further. We’re ready to support and answer questions.”* Additionally, the unit had a technology-interested nurse formally assigned as a superuser. However, she did not participate in the training. In hindsight, the unit figured out that she probably should have.

There was also some resistance to video consultations when nurses' responsibility toward patients and families and their professional knowledge was contested. One nurse said, “*We feel responsible when we send the patient on early homestay. What if the baby turns yellow* [jaundice]*? Can we see that through a screen?* […] *We cannot see whether they* [babies] *are dirty or if they* [the parents] *get them washed up and those kinds of things, so there are a lot of things we cannot see*”. On the contrary, the nurses were positively surprised about the video quality during the training session. Still, during the hands-on part of the training sessions, they learned that tasks involving bodily and sensuous impressions were not an option. They expressed that they needed to be, e.g., able to feel a hernia or the soft palate with a finger.

However, video consultations were accepted when unit management was responsive to the nurses’ concerns regarding patient safety and soundness. The responsiveness led to customization of the video consultation use; “*We have the permission to consider from time to time whether it should be a video consultation or a physical home visit. But it is okay that half of the visits are physical* […]. *We think the offer we have now, combining video consultations and physical home visits, is… They* [the families] *are safe, right?*”. The parents found the technology easy to use, which was also important to the nurses.

Despite some resistance, video consultations were used 50% of the time, and the nurses expressed the importance of deciding themselves, based on their professional knowledge, whether a consultation should be physical or on video. An added value of the video consultations was letting go of guilt due to saved time, as one nurse expressed it: “[…] *if I stayed in the ward, I could help my colleagues because we often have a bad conscience when the ward is busy. I could have had more time to help them the rest of the day, right?”.*

### Cardiology unit

The cardiology unit carries out various outpatient examinations, treatments, care, and specialist rehabilitation of patients with cardiovascular diseases. In 2020, the unit joined a project intending to use video consultations in care transitions to respond to regional and patient expectations. The unit management met with the trainers and planned the implementation and training process.

During observation of the training sessions, it was apparent that the training was conducted as in the neonatal unit. As the objective of the video consultations was care transitions, the participants consisted of staff from hospitals and the municipality. The training involved case-based exercises, where staff from the hospital and staff from municipalities were paired and should set up video consultations and discuss a case. The IT specialist who attended the training was needed as it was difficult to set up the devices from the municipalities to log in to the system used by the hospital. Like in the neonatal unit, the trainers sneaked into the training facility during lunch and turned off sound and cameras in various ways. The staff then had to troubleshoot and make their devices work again.

According to the nurses, they appreciated the training. The training was necessary, but they also said: *“I have to admit that I thought it was too smart because it didn’t even work when we did the training.”* Further, the nurses said they “*couldn’t log on to the system”* during training and did not “*get an immediate experience of success.”* One of the nurses said to get the video consultations scaled up after the training, "*They needed someone to guide them and to be present*.” As the unit did not have formal or informal superusers, they appreciated the follow-up and support from the trainers. One of the nurses said: “*The follow-up is important because when I’ve been to the training, I thought this could have been done differently”.* Despite some bumps on the road, COVID-19 forced them *“to try and see if we could start. If not, we wouldn’t have begun with video”.*

Some resistance was related to ‘the medical gaze’ and the patient relation. The head nurse said, "*It’s not about being resistant because you don’t want to do anything new. It’s about trying to see the person first”.* A nurse said video consultations “*can’t reflect empathy* […]. *What do a nurse and a physician do? It’s not only objective, you know. It’s what comes out of the mouth* […]*. It has something to do with… We sit in consultation and register signals. We do that with all our senses, including sounds and smells. It’s all. It tells me about many things”.* Another nurse agreed and said: *“Yes, I think we are really dependent on so many parameters. I have an example* […] *I had him* [a patient] *and observed that he was much worse than he thought. Because he told me he was well, and he wasn’t. It turned out at the heart scan that he was in a really bad condition. But he thinks he is well, which is what he tells me. If it were on video, I wouldn’t know. Because his pulse was way too high. One of the scars was infected. There were so many things that he didn’t react to. He did not tell me, and I could not find out. Even though I specifically asked these things, he was not capable of identifying or telling me that this was wrong because he did not know himself”.*

Further, the nurses expressed that through video consultations, *“you do not get eye contact in the same way,” “you lose some of the relations”,* and you cannot “*reach out to someone, comfort, or calm down or* […] *touch someone”.* The nurses expressed that if video consultations should be used with patients, they must make sense and be relevant. The nurses also said that they need training in personal and communication skills and how to monitor a patient behind a screen to feel safe, as well as some positive experiences, a setup for practice, and support on the spot. According to the nurses, it is something else when communicating with peers because *“you can easier concentrate on the technical stuff”* and *“there you don’t have to concentrate on those on the other side of the screen, they understand what you say, compared to patients. Because it’s from peer to peer. That is something else* […]. *It makes good sense, and I will make it work when things make sense to me”.*

A year after the initial training, the unit used video consultations weekly to supervise municipal physical therapists responsible for rehabilitation. However, the unit manager stated, "*It’s not like it’s time-saving, at least not yet. In fact, quite the opposite. It increases the possibilities you have to handle as an employee. It requires up-to-date knowledge and awareness concerning how to behave when having patients on the phone, how to behave on video, and how to behave in physical consultations.”.* Another nurse agrees: “*It’s not saving us time yet. It’s an extra task on top of everything else. It might save us some time in the long run or fewer hospital admissions. But the beds will be filled up with other patients anyways”.*

### Neurology unit

The neurology unit treats patients with neurological conditions caused by trauma, cancer, infections, and chronic and terminal conditions. These conditions often cause complex and long-term problems. As such, the unit collaborates closely with next of kin and the municipality. Whenever a complex patient is admitted, a ‘multidisciplinary consultation’ is held with next of kin and relevant municipal actors to start the rehabilitation process. These consultations would typically occur physically in the units and would often be delayed due to next of kin living far away. In line with the regional decision to strengthen cross-sectoral collaboration with technology, the unit implemented video consultations to replace the existing workflow and make rehabilitation more efficient.

Before implementation, the trainers had a preparation meeting with the head nurse, a senior consultant neurologist, and the unit manager to plan the implementation and training. Since the unit was quite busy, they did not have the time and resources to send staff off to training in simulation facilities. As such, the training was held by the trainers in the unit and became quite practice-based. The training was conducted with practical information, case-based hands-on training, troubleshooting, and questions. As the training took place in the hospital unit, municipal actors did not participate. Most of the staff in the unit participated in the training and seemed to have the needed digital skills on the spot.

However, three months after the training, the head nurse stated: *“I won’t say that it is easy to implement – it's not. The staff… Many received training in this, and I heard that when people went from the training, they said, ‘Oh, was it that easy?’, ‘That wasn’t so hard,’ and ‘We will figure that out easily.’ And then we got the iPad, and were going to start and then ‘how was it again?’, ‘I don’t remember,’ you know… And instead of spending 10 min in a busy workday, it’s easy to say, ‘I don’t have time for it today. I’ll do it another day. We succeed in using it now and then, but it really requires a short and precise manual on how to do it* […]. *It is not because they fear technology or anything… It’s just that… You tend to revert to old habits. Because that is easiest, I think. It requires persistence to say that this is what we want and this is what we do. I also experience, when we succeed, that we get feedback from next of kin where they say, ‘Oh, it is so convenient that we can do it this way,’ ‘Oh, it is so nice,’ and ‘We can see and hear that it works really well.’ I hope this kind of positive feedback can boost the implementation and get the laggards moving”*, and that: *“There has not been much resistance. Everyone can see that it makes sense in certain situations. But the thing is… To ensure that everyone maintains and uses it in these situations, and not take the line of least resistance, and then do what they normally do”.*

Unlike the other two units, there was no formal or informal follow-up and support from the trainers after the initial training course. Instead, they helped each other and used a short manual describing the process. According to the head nurse, *“They are good at helping each other. Some remember better, some just used it, and for some it’s a long time since they used it. So, they use each other.”* Initially, a secretary was formally assigned to be the unit’s superuser. However, after some time, a nurse *“with a flair for technology”* returned from sick leave and informally took over as the unit’s superuser.

Three months after the initial training, the unit had held ten multidisciplinary meetings on video with the next of kin. The unit experienced that they “*get to clarify the next steps faster, making the hospital stay shorter.”* However, according to the head nurse, they “*do not look at it as a financial saving, but as a quality boost when communicating with patients, next of kin and other collaborative actors.”* Even though the video consultations aimed to strengthen the cross-sectoral collaboration, they strengthened the involvement of next of kin as they “*want to use it where it makes sense, and where it can benefit*.”

### Summary

The nurses at the neonatal unit expressed that they acquired the needed skills to use video consultations with patients and families. However, their willingness to take responsibility for using video consultations depended on being able to decide whether a video consultation would be sufficient to satisfy their responsibility to the patients.

The cardiology unit staff needed personal and communication skills and training in monitoring a patient behind a screen to use video consultations with patients. However, they were willing to take responsibility for using video consultations with peers (once a week), where patient soundness was not contested, and the same personal and communication skills were not needed.

According to the neurology unit head nurse, the staff acquired the skills to use video consultations to communicate with the next of kin. As the patient was at the hospital, the patient's soundness was not contested. Instead, the patient soundness might have been increased as the hospital admission became more coherent by including the next of kin through video consultations. However, the neurology unit struggled to routinize video consultations and to change existing workflows as they experienced video consultations as being more time-consuming, even though they had the potential to make a hospital stay shorter. Next, we present the mechanisms and contextual factors that generated the various outcomes.

### Mechanism 1: workplace learning

#### Support on the spot

After the formal training course, the trainers offered informal support in the units whenever needed. The informal support appeared to be particularly successful in providing a feeling of safety among healthcare staff when using new technology. The need for informal support often occurred the first couple of times when healthcare professionals used the technology.

#### Continuous follow-up and focus

Formally scheduled follow-up meetings 30–60-90 and 180 days after the initial training, along with the spontaneous needs-based support, led to a continuous focus on getting the video consultations implemented and a continuous focus on ensuring that healthcare professionals had the needed competencies to use the video consultations. If the necessary competencies were lacking, healthcare professionals were frequently encouraged to continue practising (with support).

#### Establishing responsible roles

Responsible roles were either assigned formally or taken informally. If assigned formally, the roles were given to either willing and technology-interested healthcare professionals or more “senior” staff with other pressing tasks or other areas of responsibility. If the last happened and no one took responsibility, some health care professionals took an informal responsible role. As such, there were formal or informal superusers to provide support. Available support among peers lowered the threshold of testing the new video consultation system.

#### Knowledge exchange across disciplines and sectors

The formal classroom training where staff across sectors were gathered led to unity and knowledge exchange across disciplines and sectors concerning implementation challenges. However, formally scheduled video consultations with the municipality were not always coherent with the unpredictable workflows at the hospital.

#### Summary

**Fig. 1:**
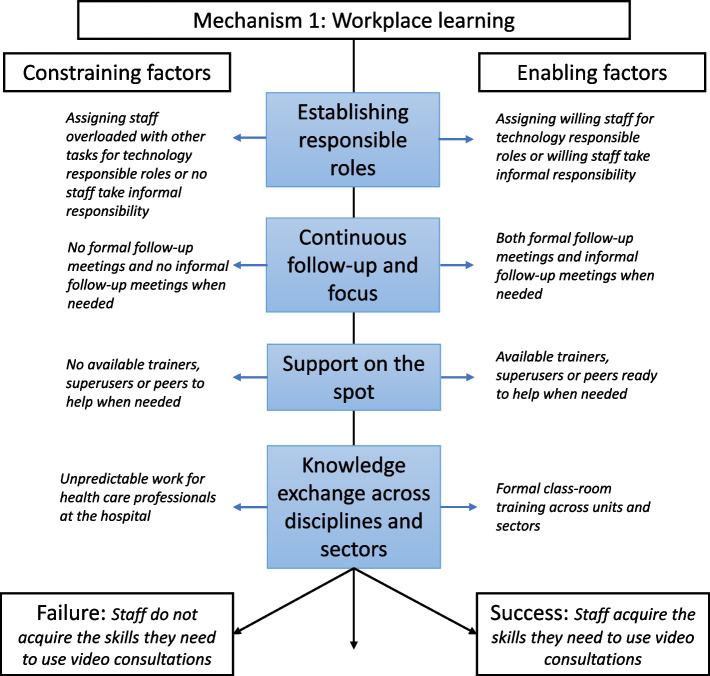
Realist analysis of attempts to implement video consultations by skills acquisition through workplace learning

Figure 1 shows a realist analysis of the enabling and constraining factors on workplace learning. The implementation experience suggests that efforts of workplace learning were more likely to succeed when there was the available staff (trainers and superusers) to provide support and to ensure a continuous focus on implementing and learning how to use video consultations in day-to-day practice. Moreover, gathering multidisciplinary and cross-sectoral staff in training sessions might lead to a common understanding of varying workflows, challenges, and opportunities.

### Mechanism 2: Professional judgment

#### Coherence with patient soundness

Healthcare professionals expressed that video consultations increased the coherence with patient soundness when the technology was well functioning with a clear image and adequate sound and when healthcare professionals could decide whether a consultation should be real-life or on video. For example, if the patient had a high-risk condition. On the contrary, if the person on the other end of the video consultation was a peer or next of kin, the patient's soundness was not contested the same way.

#### Staff takes responsibility

The staff takes an informal responsibility to use video consultations if they experience video consultations as being coherent with patient soundness and their professional knowledge, and vice versa. The staff takes less informal responsibility if they experience large clinical and administrative workloads.

#### Responsiveness to professional knowledge and skills

Units with managers responsive to health care professionals' concerns regarding patient soundness were more likely to adjust the video consultations to the existing workflows and routines. Units with managers who were unresponsive, goal-oriented, and technology-pushing managers were more likely to increase resistance among healthcare professionals. The resistance led to video consultations not being used because they were not adapted to existing workflows and were not experienced as coherent with patient soundness.

#### Summary

**Fig. 2:**
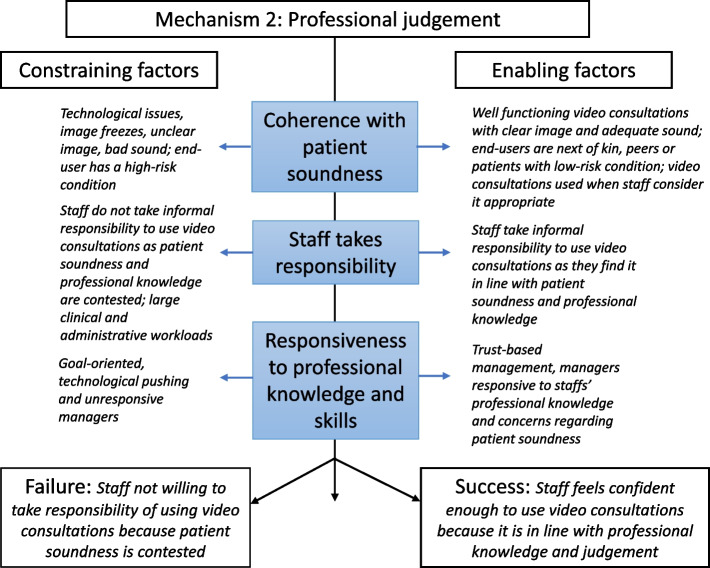
Realist analysis of implementing video consultations by adjusting to professional judgment

The realist analysis of approaches to adjusting the video consultations to workflows and routines (see Fig. 2) and not adjusting workflows and routines to the technology suggests that these efforts were more likely effective when video consultations were experienced as coherent with patient soundness. If that were the case, healthcare professionals would take responsibility for using video consultations. However, unit managers had to be responsive to and act on healthcare professionals’ concerns and decisions regarding the patients to achieve that.

## Discussion

The projected shortages in the health workforce and the changing needs of ageing populations have increased the focus on implementing video consultations [4]. Although the COVID-19 pandemic is seen as a promoting factor for video consultation use [3, 4], our study shows that skills acquisition and novel workflows are challenging [16]. Significant organizational and infrastructural challenges exist to overcome to succeed with video consultations [4, 10].

The most reported challenges are the establishment of new workflows and organizational routines and the lack of technical support, resources, and training [11, 12]. These challenges were also present in this study, showing that to succeed with implementation, there is a need for informal workplace training, continuous support, follow-up, and adjustments of video consultations to existing workflows and routines to fulfil patient soundness. In both the neonatal and cardiology units, the nurses experienced the patient soundness as contested due to a lack of sensuous and bodily impressions through video consultations, as highlighted elsewhere [17]. Alternating care delivery has been reported as one way to overcome such a barrier [12], as was the solution for the neonatal unit. However, the cardiology unit only used video consultations with peers, as they expressed a need for technical skills and communication skills to monitor a patient behind a screen.

Previous research has not been very specific regarding what training and skills healthcare professionals need to utilize video consultations. The preparation meetings in the education intervention in this study were an attempt to deliver needs-based, structured, and contextualized training, as recommended by previous research on telehealth [14, 15]. The training in the units was initially held as formal courses with information, case-based hands-on learning, troubleshooting, and questions. However, during the first rounds of interviews and participant observations, it soon became apparent that formal training was not enough to implement and routinize video consultations. Even though the staff had attended a formal training course and demonstrated that they had the needed skills at the end of the course, the trainers experienced that the staff did not use the video consultations in workplace practice. The challenges with the functionality of the video consultations the staff met during training, might have constrained their experience of patient soundness.

The initial program theory of the education intervention was that multidisciplinary collaboration between relevant stakeholders (C) should lead to a shared understanding of implementation challenges and training needs (M) and, as such, to a shared understanding of solutions (M), which in turn would lead to tailormade training (C) providing health care professionals with the skills they need to adopt and routinize video consultations (O). However, after the first round of interviews and observations, the initial program theory was refined to spending sufficient time on planning the training and implementation with relevant stakeholders (C), which leads to a better understanding of implementation challenges and training needs (M), and as such, to a better understanding of solutions and contextualized training (M). Contextualized training (C) provides healthcare professionals with the skills to use video consultations immediately after training, but not necessarily in the long run (O).

After the last round of interviews, it became apparent that the units that succeeded in implementing and routinizing video consultations succeeded through informal workplace learning, which is also supported by previous research on WPL in organizations outside healthcare, reporting that most of WPL is informal, ranging from 60–90% [20, 21]. As such, the program theory was refined one last time to: Spending sufficient time on planning the training and implementation with relevant stakeholders (C) leads to a better understanding of implementation challenges and training needs (M), and as such, to a better understanding of solutions (M), and to contextualized training (C). Contextualized training, consisting of both a formal course and informal workplace learning, in combination with follow-up and support by trainers and/or superusers (C), provides healthcare professionals with the skills and confidence they need to adopt and routinize video consultations (O).

Our study aimed to identify the contextual factors that trigger mechanisms generating workplace learning, skills acquisition and the utilization of new digital skills to use and routinize video consultations. To succeed with workplace learning and skills acquisition, the findings showed that having informal and formal responsible roles in providing informal and formal support on the spot and ongoing focus and follow-up were key sub-mechanisms. These sub-mechanisms were more or less evident in all three units, and the contextual factors that triggered these outcomes were having available trainers or formal or informal superusers ready to provide both planned follow-up and support on the spot whenever necessary. These contextual factors facilitated a continuous, dynamic, situational and informal learning process after the formal course. The neonatal unit had support and follow-up provided by trainers and a formally assigned superuser. The cardiology unit had support and follow-up provided by the trainers. An informal superuser was the only one to provide support and follow-up in the neurology unit. As apparent in the three units and in line with previous research on WPL, learning is relational, and people learn from each other to find solutions for challenges at the workplace [25–27]. Further, as in line with our findings, workplace learning is enabled by organizational support and having access to peers, expertise, and learning networks [28, 29].

All three units succeeded in acquiring the skills they needed to use video consultations as such. However, the road to success and how and with whom the video consultations were used in the units differed. The neonatal nurses experienced that they had the needed skills to use video consultations with patients and families. In contrast, the cardiology unit lacked the skills to use video consultations with patients. The cardiology unit was the only unit participating in planned classroom training across sectors, triggering the knowledge exchange mechanism across disciplines and sectors. As such, the cardiology unit was the only unit that used video consultations to communicate with peers in another sector. Due to a lack of time and resources, training in the neurology unit was held in the unit and not as a formal course in simulation facilities. As such, the training became more informal, practice-based, and contextualized compared to the other two units. Despite this customization, the unit struggled to change existing workflows and to routinize the video consultations, regardless of clear benefits.

Previous research has shown that changing existing workflows requires investments in time to train new workflows and techniques, which affects both efficiency and effectiveness, often leading to resistance [12], as evident in the neurology unit. Healthcare professionals in the neurology unit tended to revert to old habits and take the least resistant line instead of using video consultations during a busy workday and struggled to routinize the new workflow. To succeed with utilising new digital skills and routinization of video consultations, staff had to feel confident and find video consultations coherent with professional knowledge and judgment. A key mechanism to generate these outcomes was professional judgment, and sub-mechanisms were patient soundness, staff responsibility, and responsiveness to professional knowledge and skills. Previous research has recognized the same mechanisms for utilizing video consultations, pointing towards the outcomes being shaped by invisible work and negotiations of professional knowledge and responsibilities in the dynamic relationship and interactions between people and the video consultations [17]. In our study, healthcare professionals decided how and whether to use video consultations based on tacit knowledge. If managers and leaders somehow succeeded in considering, acting on and being responsive to healthcare professionals' tacit concerns and decisions regarding patient soundness, they adjusted novel workflows with video consultations according to healthcare professionals’ judgment, knowledge, and skills. These adjustments led to patient soundness and professional knowledge not being contested. As such, staff took responsibility for utilizing video consultations, and routinization was more likely.

The sub-mechanisms were evident in all units, but the contextual factors in each unit triggered varying outcomes in the three units. In the cardiology unit, the staff did not feel confident enough to use video consultations with patients. However, in the neurology unit, the patient was admitted to the hospital, still a part of the video consultation, but with next of kin on the other side of the screen. The neonatal unit succeeded in using video consultations with patients and families. However, only in combination with physical visits. The patient soundness was not contested if the technology was well-functioning and the person on the other side of the video consultation was a peer (cardiology unit), next of kin (neurology unit), or a patient with a low-risk condition (neonatal unit).

Large clinical and administrative workloads have been reported as the primary barrier to WPL [28], especially in the neurology unit, triggering less informal responsibility to use video consultations among the staff. However, workflow adjustments led staff to take more responsibility for video consultations. For example, alternating care delivery [12], as in the neonatal unit or using video consultations with peers or next of kin instead of patients, as in the other units.

### Implications for practice

Successfully implementing video consultations in healthcare settings goes beyond sufficient funding, supportive policies, and formal training. It involves recognizing the significance of informal learning processes. Video consultation training and implementation requires careful planning, including a) providing planned and informal support and continuous follow-up even after formal training and b) ensuring the availability of trainers, either through formal superusers or knowledgeable peers, for support.

Moreover, healthcare professionals often draw on tacit knowledge when deciding whether and how to use video consultations, emphasizing the importance of managers and leaders considering a) acknowledging and addressing healthcare professionals’ concerns and decisions regarding patient soundness and b) adjusting video consultations to novel workflows based on healthcare professionals’ judgments, knowledge, and skills.

### Issues for further research

This study has demonstrated the value of RE in unpacking the complexity when learning how to use and implement video consultations, which can be argued only to have been made possible by using RE. However, unpacking the complexity of skills acquisition and video consultation implementation is not a process driven solely by logical deduction. As others [40] have pointed out, unpacking mechanisms in realist evaluations is not as straightforward as described [32]. Instead, it’s an interpretive undertaking that requires extensive negotiation and debate among the researchers and moving back and forth (and back and forth) between theory and data.

Our findings showed that mechanisms are indeed underlying, often unobservable or hidden [38, 39]. Our bare eyes could detect some constraining and enabling contextual factors, but the most significant contextual factors were tacit, informal, and found between the lines.

The RE has proven beneficial to surface how contextual factors interact, constrain, and enable our study's detected mechanisms. However, we argue that the RE would benefit by being supplemented with other theories of change, such as symbolic interactionism, not immediately associated with a realist philosophy of science. Further studies could benefit from delving into social dynamics, how actors change in interaction with other actors, and how they communicate verbally and non-verbally, especially the latter, as many of the detected factors in our study were informal and found in the unsaid.

### Strengths and limitations

This study covers 18 months of real-world training and implementation, including interviews and observations of a relatively high number of participants. Using RE and CMO configurations to figure out ‘what works, for whom, under what circumstances, and why’ has been a useful approach and has provided us with a deeper understanding of contextual factors and mechanisms that enable workplace learning, skills acquisition, and utilization of digital skills to use video consultations.

Still, this research is not without limitations. Transferability to other fields may be challenging since the findings build on a comparative case study. However, the rich case descriptions may enable readers to determine transferability to other fields.

As mentioned, identifying mechanisms, contextual factors, and outcomes are not straightforward. However, the cross-case comparisons helped determine how workplace learning and professional judgment played out in the different units and produced the same or different outcomes. Yet, we identified that a constraining factor in one context might be an enabling factor in another. Further, it was sometimes challenging to distinguish between the education intervention and the contextual factors. For example, to decide whether the ongoing informal support in the units was a part of the education intervention itself or a result of the contextual factors. In some situations, the education intervention might co-construct the context and intertwine with the mechanisms.

## Conclusions

A formal training course alone is insufficient to provide healthcare professionals with the skills needed to use video consultations in workplace practice. Yet, a formal training course in combination with informal workplace learning with formal and informal support on the spot and continuous follow-up seems to acquire the needed skills among healthcare professionals to use video consultations.

The study findings contribute to practice by demonstrating that skills acquisition depends on factors such as having available trainers and informal and formal superusers in responsible roles to provide support on the spot. In addition, the findings also contribute to the concept of workplace learning, showing that providing continuous follow-up and focus by having both formal and informal follow-up meetings enables workplace learning. Moreover, a formal training course across sectors can enable knowledge exchange across disciplines and sectors, which in turn may generate collaboration across sectors through video consultations. However, unpredictable work at the hospital inhibits this mechanism.

Furthermore, the research contributes to the telehealth field by demonstrating that utilizing new digital skills and routinization of novel workflows with video consultations are more likely to succeed if unit management is responsive and if video consultations are adjusted to professional judgment, knowledge, and skills, as well as concerns regarding patient soundness. Nevertheless, these adjustments depend on contextual factors dependent on each other. The patient soundness is not contested if there is an option to alternate care delivery, if the end-user is next of kin, peers, or a patient with a low-risk condition, and if the video consultations function well. If the patient soundness is contested, healthcare professionals do not take an informal responsibility to use video consultations. Further, large clinical and administrative workloads constrain informal responsibility. However, if unit management is responsive to and adjusts the video consultations to these sometimes informal and invisible contextual factors, healthcare professionals are more likely to feel confident to use and routinize video consultations.

### Supplementary Information

**Additional file 1. **Interview guide for semi-structured focus groups and individual interviews.

**Additional file 2. **Observation guide.

## Data Availability

The data collected for this study consists of transcribed interviews and field notes. These qualitative data will not be made available due to privacy reasons. An interview guide (additional file 1) and an observation guide (additional file 2) are available.

## Abbreviations

CMO: Context-Mechanism-Outcome
RE: Realist evaluation
WPL: Workplace learning
