# Supplementary material for: The informal way to success or failure? Findings from a comparative case study on video consultation training and implementation in two Danish hospitals
Source: BMC Health Serv Res. 2023 Oct 21;23:1135. doi: 10.1186/s12913-023-10163-w (PMC10590501; doi:10.1186/s12913-023-10163-w)
Supplement: Supplementary file 1 — Additional file 1. Interview guide for semi-structured focus groups and individual interviews. [file 12913_2023_10163_MOESM1_ESM.pdf]

# Interview guide

**The interview starts with questions related to participant characteristics (around the table if focus group):** Can you describe the tasks and responsibilities you have in your workplace? How long have you had your current position? What is your professional background? What is your age?

**Questions:**

1. Can you tell me about the video consultations you have been trained in or use during your workday?

2. What has been the greatest success when it comes to the implementation and use of telehealth technology in the unit? Can you tell me about a project or collaboration that went well? What happened? Why did it work so well? Can you tell me about a project that did not go so well?

3. Do you use video consultations during your workday? (Why not?) In what way do video consultations affect your workday?

4. What is the biggest value of using video consultations?

5. What is the biggest challenge o fusing video consultations?

6. What do you do if the video consultations do not work as they should?

7. Who do you collaborate with when you use video consultations? How do you collaborate?

8. Have you been involved in the planning of the implementation of the video consultations, or the planning of the training? How have you been involved?

9. Have you participated in any of the planning meetings? How did you experience that? What worked? What did not work? Do you have any suggestions for changes?

10. How did you experience the video consultation training? How did it take place? What worked? What did not work so well? Do you have any suggestions for changes?

11. In what way has the training influenced the way you use video consultations?

12. Have you gone through any form of evaluation after the training? How did the evaluation take place?

13. How is the atmosphere in the organization or the hospital unit? Is there anything particular influencing the use of video consultations positively or negatively?

14. How does the management create an environment that is enabling or constraining the use of video consultations?

15. What kind of support do you get within the hospital or outside the hospital that makes a difference in the implementation and the use of video consultations?

16. We would like to end the interview by asking; What does it take to succeed with the implementation of video consultations? What is the key to success? Or the opposite? What can others learn from your experiences?

17. Is there anything else you would like to add?
